# Recurrent locally uncontrolled infection in endocarditis, a fearful complication

**DOI:** 10.1093/ehjimp/qyae117

**Published:** 2025-02-18

**Authors:** Axel Abel Rodriguez-Mendez, Hugo Gerardo Rodriguez-Zanella, Ana Maria Coeto-Cano, David Jacobo Sanchez-Amaya, Daniel Manzur-Sandoval

**Affiliations:** Cardiology Fellowship, Universidad Nacional Autonoma de Mexico, Av. Universidad 3004, Copilco Universidad, 04510, Mexico City, Mexico; Clinical Cardiology, National Institute of Cardiology Ignacio Chavez, Juan Badiano 1, Belisario Dominguez Secc 16, Tlalpan, 14080 Mexico City, Mexico; Laboratory of Echocardiography, National Institute of Cardiology Ignacio Chavez, Juan Badiano 1, Belisario Dominguez Secc 16, Tlalpan, 14080 Mexico City, Mexico; Clinical Cardiology, National Institute of Cardiology Ignacio Chavez, Juan Badiano 1, Belisario Dominguez Secc 16, Tlalpan, 14080 Mexico City, Mexico; Laboratory of Echocardiography, National Institute of Cardiology Ignacio Chavez, Juan Badiano 1, Belisario Dominguez Secc 16, Tlalpan, 14080 Mexico City, Mexico; Cardiology Fellowship, Universidad Nacional Autonoma de Mexico, Av. Universidad 3004, Copilco Universidad, 04510, Mexico City, Mexico; Cardiovascular Critical Care Unit, National Institute of Cardiology Ignacio Chavez, Juan Badiano 1, Belisario Dominguez Secc 16, Tlalpan, 14080 Mexico City, Mexico

**Keywords:** echocardiography, infective endocarditis, uncontrolled infective complication, salvage heart transplant

A 47-year-old male with a history of recurrent endocarditis, with the first event related to bicuspid aortic valve, with aortic and mitral valve replacement in 2015, and with a commando surgical procedure in 2023, presented in 2024 with a new episode of early recurrent endocarditis, involving both aortic and mitral valve prostheses. He underwent a redo commando surgical procedure. The patient received long-term antibiotic therapy guided by culture, targeting *Staphylococcus epidermidis*.

A few months later, the patient presented with acute pulmonary oedema and was diagnosed with a locally uncontrolled infection. An heterogeneous vegetation measuring 24 × 14 mm with an aortic abscess involving the mitral annulus and the mitroaortic junction (*Figure 1A*; Supplementary data online, *Video S1*). There was also dehiscence of the aortic prosthetic valve, an aortic pseudoaneurysm (*Figure 1B* and C; Supplementary data online, *Video S2*), and a fistula between the left ventricular outflow tract and the left atrium (*Figure 1D*; Supplementary data online, *Video S3*).

**Figure 1:**
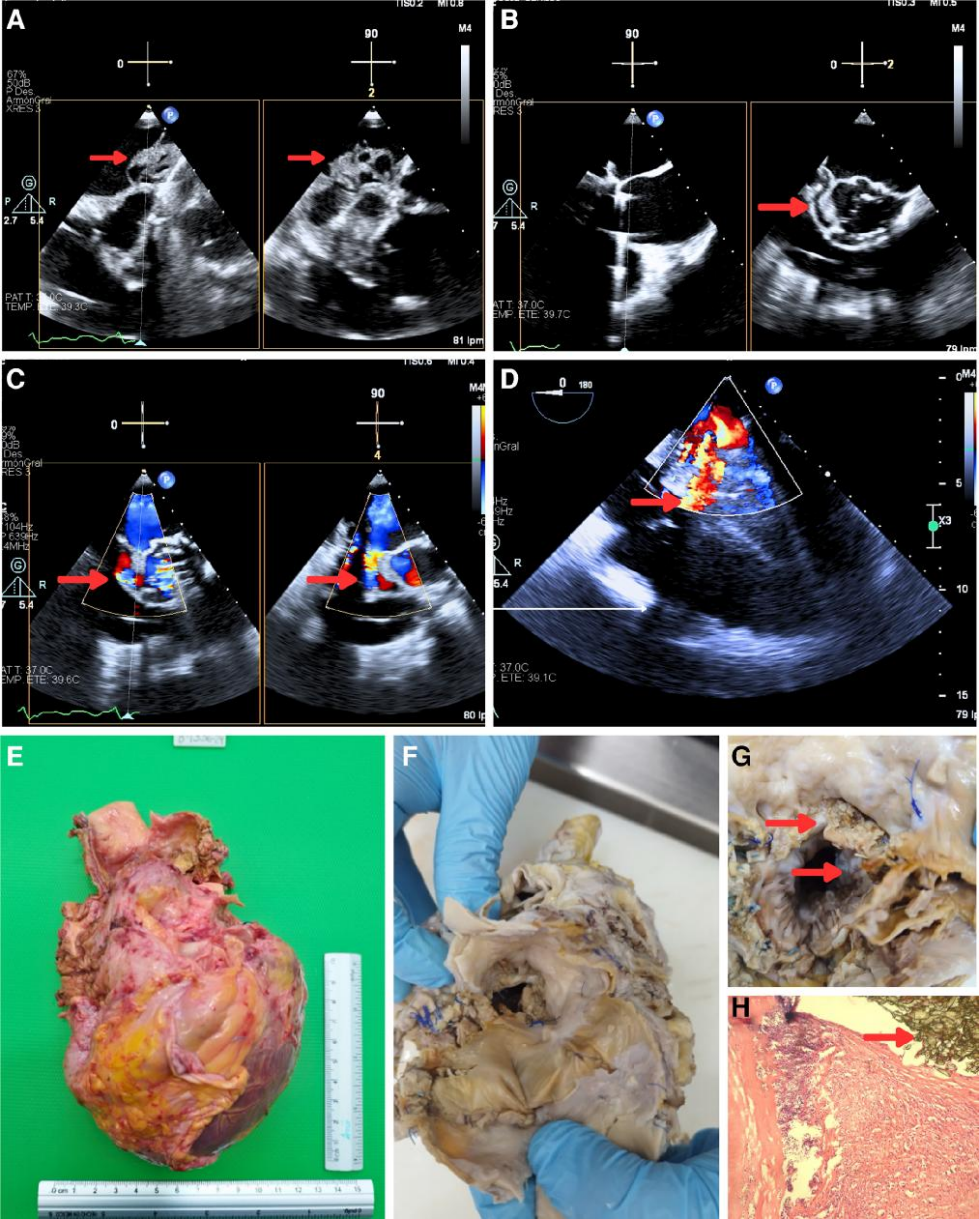


After a heart team discussion, the patient was sent for salvage heart transplant.

Examination of the explanted heart confirmed imaging findings, pericardial adhesions (*Figure 1E*), aneurysmatic dilation of the ascending aorta, and calcification of the aortic leaflets (*Figure 1F*). Additionally, there were vegetations on the aortic annulus and at the mitroaortic junction (*Figure 1G*). A microscopic examination also showed an abscess on the aortic annulus (*Figure 1H*). After, the patient suffered a fatal outcome early in the post-transplant period.

Prosthetic valve endocarditis is a serious condition often linked to complications and significant risk of life-threatening events, emphasizing the need for careful monitoring and timely intervention.

## Supplementary Material

qyae117_Supplementary_Data

## Supplementary data

**Consent:** In this case, the patient has passed away, and it is not possible to trace the patient's relatives, as they are from a different state. Therefore, we believe there is insufficient patient identification information in the manuscript.

**Funding:** The research was conducted within Instituto Nacional de Cardiologia Ignacio Chavez. No external funding was received for this study.

**Data availability:** We would like to clarify that no new data was generated or analysed specifically for this research during the course of preparing the manuscript. The data supporting the findings of this case report are available upon request from the corresponding author. Request for data should be addressed to Axel Abel Rodriguez-Mendez at axel.rodriguezmn@gmail.com.

## Lead author biography

Axel Abel Rodriguez-Mendez graduated in Medicine from the University of Sonora. He completed a specialization in Internal Medicine at the Hospital Civil in Guadalajara, Mexico. Currently, the author is pursuing a fellowship in Cardiology at the Instituto Nacional de Cardiologia Ignacio Chavez, Mexico.

